# Dinoflagellate Proton-Pump Rhodopsin Genes in Long Island Sound: Diversity and Spatiotemporal Distribution

**DOI:** 10.3390/microorganisms12030628

**Published:** 2024-03-21

**Authors:** Huan Zhang, Kelly J. Nulick, Zair Burris, Melissa Pierce, Minglei Ma, Senjie Lin

**Affiliations:** 1 Department of Marine Sciences, University of Connecticut, Groton, CT 06340, USAsenjie.lin@uconn.edu (S.L.); 2 State Key Laboratory of Marine Environmental Science, College of Ocean and Earth Sciences, Xiamen University, Xiamen 361102, China

**Keywords:** dinoflagellate, proton pump rhodopsin, spatial/seasonal variation, qPCR, LIS, temperate estuary

## Abstract

Microbial proton-pump rhodopsin (PPR)-based phototrophy, a light-harvesting mechanism different from chlorophyll-based photosystems, may contribute significantly to solar energy entry into the marine ecosystem. PPR transforms solar energy into cellular energy that is used for various metabolic processes in the cells or flagellar movement. Although rhodopsins or their encoding genes have been documented in a wide phylogenetic range of cultured dinoflagellates, information is limited about how widespread and how spatiotemporally dynamical dinoflagellate PPR (DiPPR) are in natural marine ecosystems. In this study, we investigated DiPPR in Long Island Sound (LIS), a temperate estuary of the Atlantic Ocean between Connecticut and Long Island, New York, USA. We isolated six novel full-length dinoflagellate proton-pump rhodopsin cDNAs, expanding the DiPPR database that is crucial to PPR research. Based on these new sequences and existing sequences of DiPPR, we designed primers and conducted quantitative PCR and sequencing to determine the abundance and diversity of DiPPR genes spatially and temporally throughout a year in the water samples collected from LIS. DiPPR genes were found year-round and throughout LIS but with higher abundances in the eutrophic Western Sound and in April and July. The gene diversity data suggest that there are at least five distinct rhodopsin-harboring groups of dinoflagellates throughout the year. The abundance of DiPPR genes, measured as copy number per mL of seawater, appeared not to be influenced by water temperature or nitrogen nutrient concentration but exhibited weak negative correlations with orthophosphate concentration and salinity and a positive correlation with the abundance of DiPPR-harboring dinoflagellates. This first quantitative profiling of PPR in natural plankton reveals the prevalence and dynamics of this plastid-independent photoenergy harvesting mechanism in a temperate estuary and provides efficient DiPPR primers potentially useful for future research. Furthermore, this study shed light on the potential role of DiPPR in phosphor nutrition and dinoflagellate population, which warrants further studies.

## 1. Introduction

Prior to the year 2000, chlorophyll-based photosynthesis had been considered the sole mechanism by which aquatic organisms draw energy from sunlight. Marine phytoplankton contribute about 50% of global photosynthetic productivity [1,2]. However, with the discovery of proton-pump rhodopsin (PPR) in marine bacteria in 2000 [3,4], it has become clear that rhodopsin-based phototrophy is responsible for capturing a substantial portion of the solar energy that enters the marine ecosystem daily [5,6,7]. PPRs are now found in various archaea, bacteria, fungi, and algae (e.g., [3,4,8,9,10,11]). With few exceptions, PPRs transform solar energy into cellular energy, which can support various cellular functions such as ATP synthesis, substrate transportation, and survival of bacteria under carbon starvation [6,9]. Metagenomics data show that 13–60% of the bacterial genomes in surface ocean possess PPR genes [12,13,14,15,16]. Since each rhodopsin binds a single molecule of retinal, the total number of retinal molecules is equivalent to that of the rhodopsins. Gómez-Consarnau et al. [7] quantified the vertical distributions of the all-*trans* retinal and two other energy-converting pigments, chlorophyll *a* and bacteriochlorophyll *a*, along a nutrient gradient in the Mediterranean Sea and the Atlantic Ocean. They discovered that PPR-based phototrophy likely contributes the same amount of light energy fixation as chlorophyll *a*-based phototrophy does and that the energy obtained by PPRs is sufficient to support the basal metabolism of bacteria in the surface ocean. Their study provides quantitative evidence that PPR phototrophy is a major light energy harvesting mechanism in the surface ocean.

**Figure 1:**
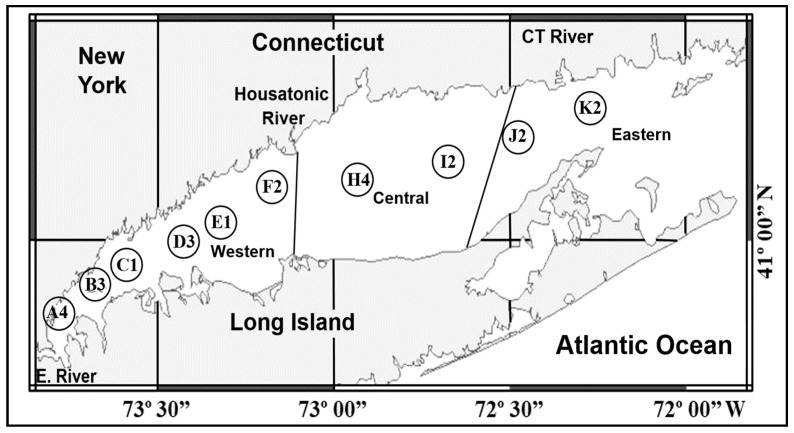
Sampling stations in Long Island Sound. A4, B3, C1, D3, E1, F2, H4, I2, J2, K2: stations where the water samples were collected.

Among the rhodopsin-harboring microbial eukaryotes, PPR genes seem to be most widespread in dinoflagellates, a group of unicellular eukaryotes characterized by various unusual genomic and physiological features [10,17]. Initially detected in *Pyrocystis lunula* Schütt through microarray analysis [18], later through transcriptomic studies on laboratory cultures, PPR genes have been documented in basal lineages such as the heterotrophic lineage *Oxyrrhis marina* Dujardin [10,19,20], autotrophic lineages *Polarella glacialis* Montresor, Procaccini, and Stoecker [8], *Prorocentrum shikokuense* Hada (=*P*. *donghaiense* Lu) [21], the mixotrophic lineage *Karlodinium veneficum* Larsen ([22] and this study), to the evolutionarily most recent lineage *Alexandrium* spp. [10]. Except those from *P. shikokuense* and *K. veneficum*, most of the known dinoflagellate PPR genes (referred to as **typical dinoflagellate PPR**, or **DiPPR** hereafter) share high similarity to PPRs from the gamma-proteobacteria of the SAR86 group [3]. We previously detected two DiPPR cDNAs from a metatranscriptome of a natural plankton assembly [8], and there are some data generated from dinoflagellate blooms [23,24], diatom-dominated community [11], or general microbial eukaryotes [5] are available. However, thus far, no studies have been dedicated to investigating the spatial and seasonal variation of DiPPR gene diversity and abundance in the field. To fill the gap of quantitative data for the spatial and seasonal variation of the DiPPR gene in the field, in this study, we chose Long Island Sound (LIS) as our study location. LIS was chosen because it is a highly urbanized estuary. Due to the large population and intensive development in the closest city, New York City, the Sound is subject to high loadings of nitrogen and phosphorus from both wastewater treatment plants and non-point sources. There is a stronger impact in the western Sound due to its proximity to New York City compared to the relatively pristine eastern Sound, and persistent episodes of summer hypoxia pose a significant environmental hazard for benthic species, including the commercially important American Lobster *(Homarus americanus* H. Milne-Edwards) (https://portal.ct.gov/-/media/DEEP/water/lis_water_quality/monitoring/2020/2020-LIS-Combined-Report.pdf) (accessed on 14 March 2024). In addition, located at a latitude of 41° N, LIS represents a typical temperate estuary, with strong seasonal cycles of temperature and irradiance that drive classical spring bloom and summer stratification phytoplankton communities. We developed inclusive DiPPR primers and isolated six full-length DiPPR cDNAs from the water sample collected at the Avery Point campus of the University of Connecticut located at the eastern end of LIS close to Station K2 (Figure 1) and two full-length rhodopsin cDNAs from *K. veneficum*. We conducted quantitative PCR to analyze the spatial and temporal variations of the abundance and diversity of DiPPR genes over the course of one year in the water samples collected along a eutrophication gradient (from east to west) of LIS.

## 2. Materials and Methods

### 2.1. Water Sample Collection

Water samples were collected by the personnel of the Long Island Sound (LIS) Water Quality Monitoring Program from the Connecticut Department of Energy and Environmental Protection. Surface water sampling was carried out each month from January to December 2010 from ten stations (A4, B3, C1, D3, E1, F2, H4, I2, J2, and K2) except when inclement weather prevented cruises (Figure 1). Two hundred ml of water samples were collected 2 m below the water surface. For the preservation of phytoplankton cells, four mL of neutral Lugol’s (Utermöhl’s) solution were added to each water sample (final concentration 2%) as reported [25].

A total of 95 water samples were collected (Table 1). The preserved samples were kept at 4 °C in darkness until processing, generally within 2 months. For each sample, a 50 mL subsample was concentrated using a Utermӧhl Settling Chamber for 48 h. The supernatant was aspirated so that the sample was concentrated to 1 mL and examined using an Olympus BX51 microscope (Evident Scientific, Waltham, MA, USA) equipped with 10 and 40× magnification lenses on the objective and 10× in the eyepiece to achieve up to 400× magnification. Phytoplankton were identified to the lowest taxonomic level possible, usually to the genus level. The detailed procedures of cell counting and taxonomic verifications were presented in the Final Report for our 2010 CT DEEP grant entitled “Identification of Phytoplankton collected from Long Island Sound” and is available upon request. A parallel set of the samples was taken for genomic DNA (gDNA) extraction.

### 2.2. Genomic DNA Extraction

Genomic DNA was extracted from LIS water samples according to [26] with some modifications. Briefly, for each water sample, a 50 mL subsample was concentrated to 1 mL as mentioned above, transferred to a 2 mL microtube, and centrifuged at 15,000× *g* for 2 min to pellet the cells. After the supernatant was removed carefully, approximately 100 mg of 0.5 mm zirconia/silica beads (Biospec Products, Bartlesville, OK, USA) were added to the pellet and bead-beaten at 6 m/s for 30 s using an MP Fast Prep-24 Tissue and Cell Homogenizer (MP Biomedicals, Solon, OH, USA).

Next, 250 μL CTAB buffer (2% Cetyl Trimethyl Ammonium Bromide, 100 mM Tris-HCl pH8, 20 mM EDTA pH8, 1.4 M NaCl, 0.2% β-mercaptoethanol, 0.1 mg/mL Proteinase K) was added, and the samples were incubated for 4 h at 55 °C with gentle mixing. For each sample, 250 μL chloroform was added and mixed well, centrifuged for 10 min at 15,000× *g*, and the supernatants transferred to a new 2 mL microtube. The genomic DNA was then purified using the DNA Clean & Concentrator Kit (Zymo Research, Irvine, CA, USA) and eluted in 200 μL of 10 mM Tris-HCl (pH8), making the final amount of DNA per 4 μL equivalent to the DNA from 1 mL water sample. DNA quantity and quality were determined using a NanoDrop Spectrophotometer ND-1000 (NanoDrop Technologies, Wilmington, DE, USA) and stored at −20 °C until further analysis.

### 2.3. Development of Specific Primers for Typical Dinoflagellate Proton-Pump Rhodopsin (DiPPR) Genes and Quantitative Real-Time Quantitative PCR (qPCR)

Based on the alignment of the sequences of typical DiPPR sequences available in GenBank databases and the six full-length DiPPR cDNAs we obtained in this study, several conserved regions of DiPPR were identified, and primers were designed in these regions using Primer Premier 6.0 (Table 2). These primers were tested against the cDNAs/genomic DNAs (gDNAs) of the dinoflagellates we obtained in the previous study (see [27,28] for details). The cDNA and gDNA from eight non-dinoflagellate phytoplankters were isolated in this study as the negative controls to test the specificity of the primers (Table 3). Many of these phytoplankton species were commonly found in LIS (https://www.nfwf.org/sites/default/files/finalreports1/1401.10.023622_Phytoplankton_Guide.pdf) (accessed on 14 March 2024).

Primer set DinoRhodF3N-DinoRhodR3N was found to be the most sensitive and specific to amplify DiPPR for many dinoflagellates that are known to possess typical DiPPR (Table 3). To assess spatial and temporal dynamics of rhodopsin gene abundance, Real-Time qPCR was performed as reported [29] using 4 μL of the extracted DNA as the template with primer set DinoRhodF3N-DinoRhodR3N. A dilution series of the PCR product of *Alexandrium catenella* (formerly *A. fundyence*) rhodopsin cDNA was used as a standard to calculate the copy number of this gene in LIS DNA samples, as reported [26].

In the previous study, we designed universal primers that could amplify the majority of the eukaryotic 18S rRNA gene (18S rDNA) [26]. As a reference gene with which to normalize rhodopsin gene abundance in phytoplankton communities, we also performed Real-Time qPCR for the 18S rDNA in all 95 samples using the previously reported universal primer set 18SCOMF-18SCOMR [26]. This primer set has been verified to be able to amplify all the major phytoplankton.

### 2.4. DiPPR Gene Cloning and Sequencing of the LIS Water Samples

Six DNA samples were chosen for cloning and sequencing of the DiPPR genes, including three samples from station A4 (during February, April, and August, sample # 11, 31, and 56, respectively), one sample from station E1 in April (sample # 35), and two samples from station H4 (February and August, sample # 7 and 61). To measure rhodopsin diversity in these samples, amplicons from the 2nd round of rhodopsin Real-Time qPCR were cloned into T-vectors, following our previously reported method [25]. For each sample, 48 colonies were randomly chosen, and plasmid DNA was isolated and sequenced as reported [25].

### 2.5. RNA Extraction, cDNA Synthesis, and Full-Length cDNA Isolation of DiPPR in the Field and from Karlodinium Veneficum

Water samples were collected in February 2011 at the Avery Point campus of the University of Connecticut (41°18′55″ N, 72°03′48.6″ W). One liter of water was filtered onto a 3-μm Nuclepore filter membrane, which was then immediately immersed in 1 mL TRIzol RNA buffer (ThermoFisher Scientific, Waltham, MA USA). In parallel, ~1 million cells of *K. veneficum* CCMP1975 were harvested from a culture grown on f/2 medium by centrifuging at 3000× *g* and fixing the cell pellet in 1 mL TRIzol RNA buffer. Total RNA was extracted following the reported method [19]. First-strand cDNA was synthesized and purified; full-length rhodopsin cDNAs were obtained following the reported DinoSL-based method [19] using the DiPPR primers pared with DinoSL or Racer3 primer (Table 2).

### 2.6. Phylogenetic Analysis

The sequences of the proton-pump rhodopsins in the marine bacteria and microbial eukaryotes reported in GenBank were obtained by BLAST search; the accession numbers of the representatives of these rhodopsin genes were GU553957, GU554267, X70290, X70291, X70292, AE004437.1, AF508258, GQ402542, FJ545222, L05603, Z35086, AF279106, AF135863, AY882440, DQ074124, DQ133531, CP001291, EF100190, DQ088848, EF107103, DQ088869, DQ062254, DQ088860, EF100191, DQ073796, AF349981, HQ654769, EF134313, EF134318, EF134314, EF134312, HQ654766, HQ654763, HQ654764, KF651052, KF651053, KF651054, KF651055, KF651056, HQ654767, GU138075, DQ133530, HM231308, HM231309, KM282617, and AB074484. The rhodopsin nucleotide sequences we obtained in this study and some of the representatives acquainted from the GenBank database were aligned using ClustalW [30] in MEGA X. Model in MEGA X was used to find the best model of nuclear acid evolution. Phylogenetic trees were inferred using the Maximum likelihood method in MEGA X with rates estimated from the Model in MEGA X. The evolutionary history was inferred by using the Maximum Likelihood method with the Hasegawa–Kishino–Yano model [31]. The bootstrap consensus tree inferred from 1000 replicates was taken to represent the evolutionary history of the taxa analyzed [32]. Branches corresponding to partitions reproduced in less than 50% of bootstrap replicates were collapsed. The percentage of replicate trees in which the associated taxa clustered together in the bootstrap test (1000 replicates) were shown next to the branches [32]. Initial tree(s) for the heuristic search were obtained automatically by applying Neighbor-Joining and BioNJ algorithms [33] to a matrix of pairwise distances estimated using the Maximum Composite Likelihood (MCL) approach and then selecting the topology with superior log likelihood value. A discrete Gamma distribution was used to model evolutionary rate differences among sites (5 categories (+G, parameter = 0.4893)). This analysis involved 202 nucleotide sequences. All positions containing gaps and missing data were eliminated (complete deletion option). There were a total of 230 positions in the final dataset. Evolutionary analyses were conducted in MEGA X (v.1.0) [34]. The resulting tree file from MEGA X was then uploaded to Evolview [35] to make further modifications.

### 2.7. Statistical Analyses of DiPPR/18S rDNA Gene Abundance and Relationships with Environmental Factors

To examine the potential correlation between the DiPPR/18S rDNA gene abundance and environmental factors, we obtained the monthly data for the 10 stations, including salinity, water temperature, chlorophyll *a*, concentration of ammonia, nitrate + nitrite, orthophosphate, total dissolved nitrogen and total dissolved phosphorus in 2010 (http://lisicos.uconn.edu/dep_portal.php (accessed on 14 March 2024), Figure S1). The statistical analyses of the relationship between environmental factors and the corresponding gene abundances of DiPPR and 18S rRNA in each sample were carried out using the built-in Regression function in Microsoft Office Excel 2016 Data Analysis.

## 3. Results

### 3.1. Temporal and Spatial Variations of Phytoplankton Cell Abundance

**Figure 2:**
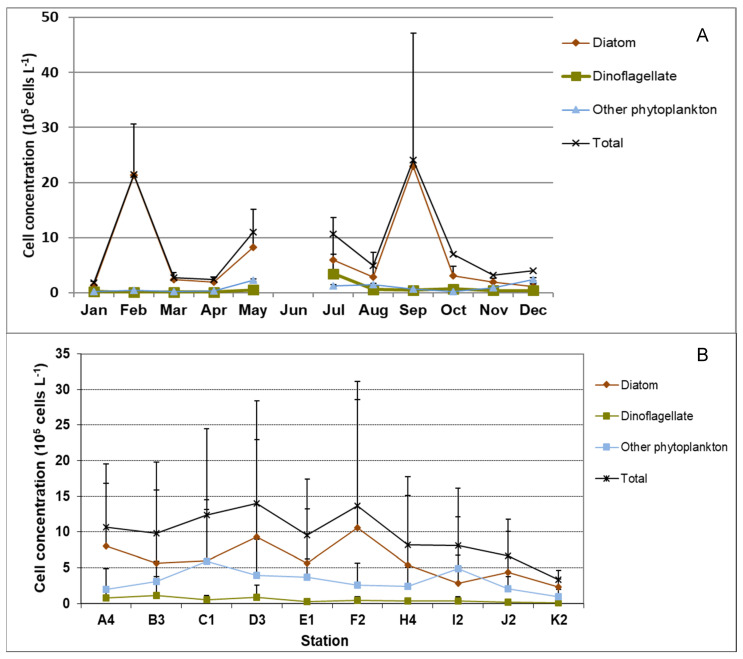
Temporal and spatial dynamics of Long Island Sound (LIS) phytoplankton in 2010. (**A**) Monthly average of phytoplankton cell concentration of diatom, dinoflagellate, other phytoplankton, and total phytoplankton for all stations in LIS. (**B**) Annual average of phytoplankton cell concentration of diatom, dinoflagellate, other phytoplankton, and total phytoplankton in each station.

The phytoplankton community showed large temporal and spatial changes (Figure 2 and Figure S2A). The average cell abundance showed two peaks, one in February and the other in September. The lowest cell abundance was found in April. Diatoms dominated throughout the whole year, while dinoflagellate cell concentration was highest in July, with the most abundant species from the genera *Gymnodinium*, *Amphidinium*, *Heterocapsa*, *Prorocentrum*, and *Scrippsiella.* The other phytoplankton, including silicoflagellates, cryptophytes, ochrophytes, haptophytes, euglenozoa, and unidentified small flagellates and nanoplankton reached minor peaks in May and December (Figure 2A, Table S1).

Annual average phytoplankton abundance was generally higher in the western LIS than in the eastern Sound. Overall, cell concentrations were higher at stations A4-F2 than at stations east of them (H4-K2). Annual average cell concentrations for stations in the western Sound (A4 to F2) were 9.6–14.02 × 10^5^ cells·L^−1^, those in the central Sound (H4, I2) were 8.1–8.2 × 10^5^ cells·L^−1^, and those in the eastern Sound (J2, K2) were 3.3–6.6 × 10^5^ cells·L^−1^(Figure 2B). 

### 3.2. Seasonal Changes of Gene Abundance of DiPPR and Phytoplankton 18S rDNA in LIS

**Figure 3:**
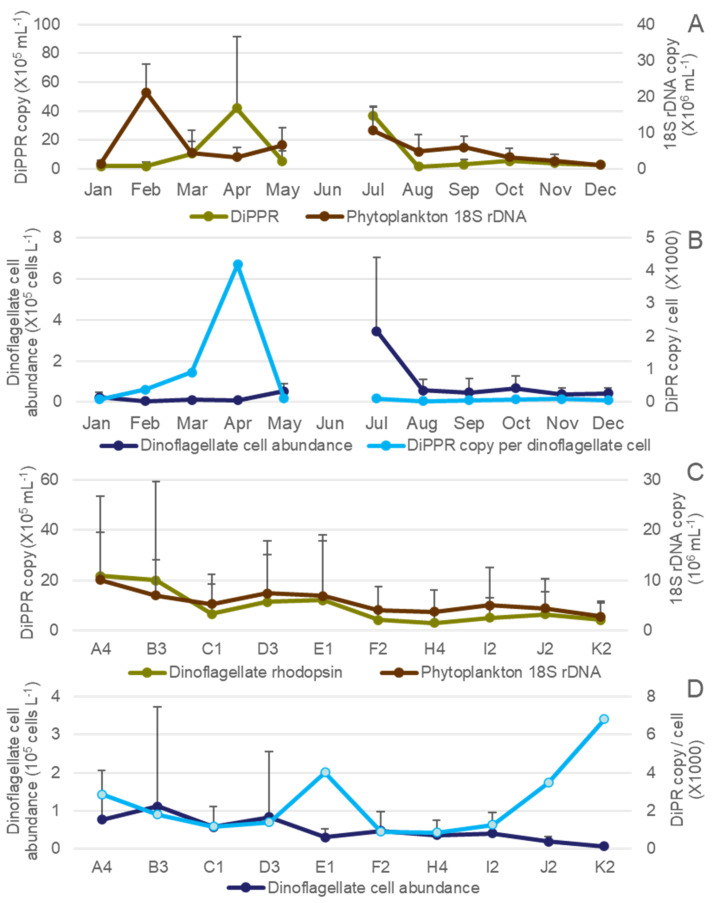
Temporal and spatial profiles of dinoflagellate proton-pump rhodopsin (DiPPR) and phytoplankton 18S rDNA gene abundance in 2010, LIS. (**A**) Temporal profile of average DiPPR and phytoplankton 18S rDNA gene abundance; (**B**) temporal profile of average dinoflagellate cell abundance and DiPPR copy per cell; (**C**) spatial profiles of average DiPPR and phytoplankton 18S rDNA gene abundance; (**D**) spatial changes of average dinoflagellate cell abundance and DiPPR copy per cell.

**Figure 4:**
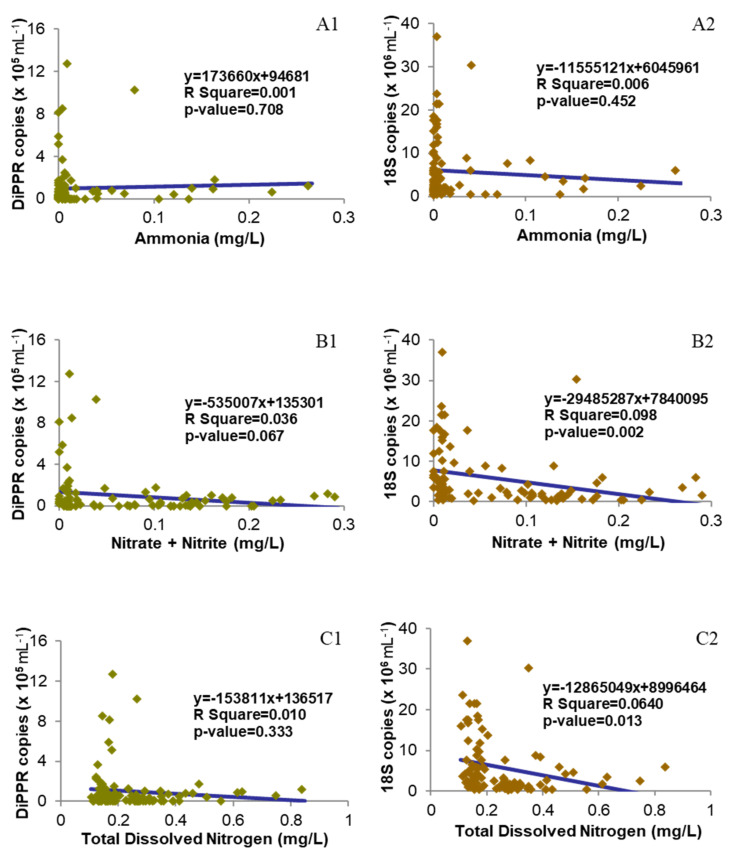
Scatter charts of gene copy numbers of dinoflagellate proton-pump rhodopsin (DiPPR; 1) or phytoplankton 18S RNA (2) vs. environmental factors with the best-fit line. (**A1**,**A2**) Ammonia; (**B1**,**B2**) nitrate + nitrite; (**C1**,**C2**) total dissolved nitrogen.

**Figure 5:**
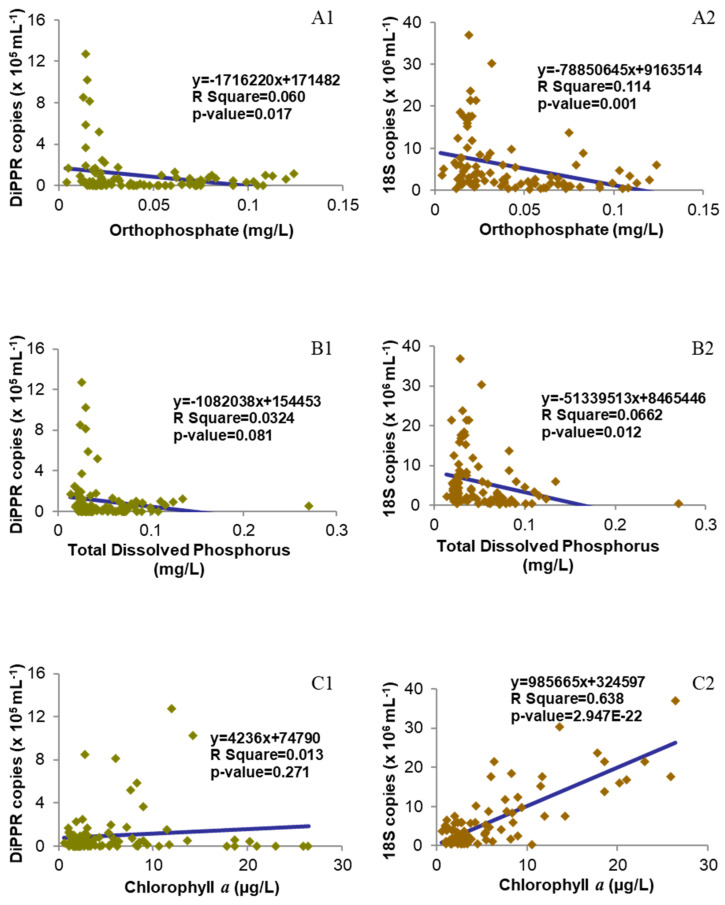
Scatter charts of gene copy numbers of dinoflagellate proton-pump rhodopsin (DiPPR; 1) or phytoplankton 18S RNA (2) vs. environmental factors with the best-fit line. (**A1**,**A2**) Orthophosphate; (**B1**,**B2**) total dissolved phosphorus; (**C1**,**C2**) chlorophyll *a*.

**Figure 6:**
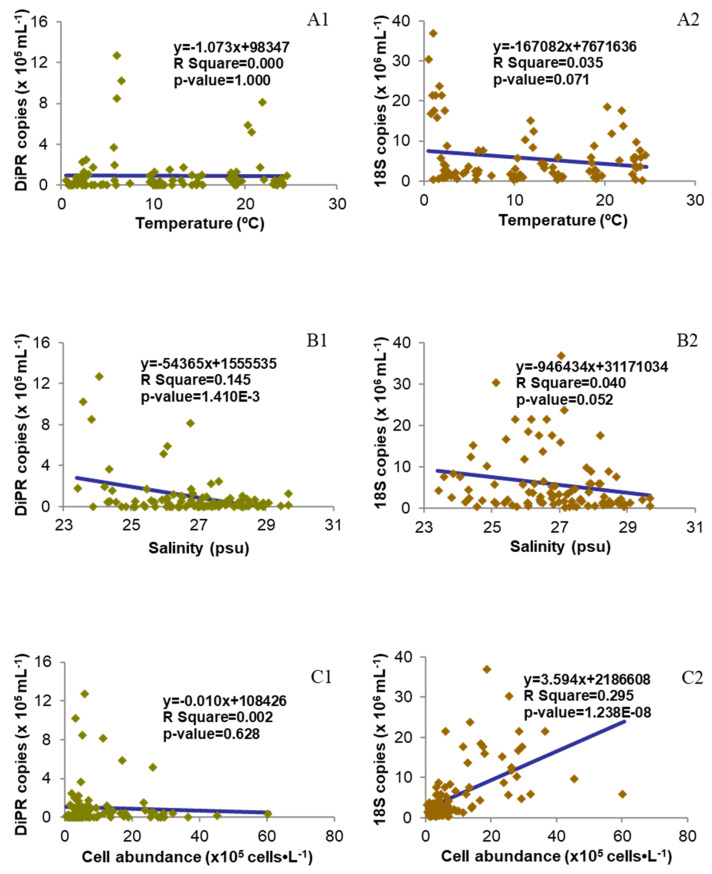
Scatter charts of gene copy numbers of dinoflagellate proton-pump rhodopsin (DiPPR; 1) or phytoplankton 18S RNA (2) vs. environmental factors (or cell abundance) with the best-fit line. (**A1**,**A2**) Temperature; (**B1**,**B2**) salinity; (**C1**,**C2**) total phytoplankton cell abundance.

Genomic DNA was isolated successfully from all 95 water samples obtained and used for Real-Time qPCR. The DiPPR gene was detected at every station with abundance ranging from several copies to up to 1.3 million copies·mL^−1^ (Figure 3A and Figure S2B), and the average monthly abundance showed two major peaks in April and July; however, when DiPPR gene abundance was normalized to the monthly average dinoflagellate cell number, only the April peak remained (4.18 × 10^4^ copies/cell; Figure 3B). The total phytoplankton18S rDNA abundance ranged from 265 thousand copies to over 36.9 million copies·mL^−1^, with a major peak in February and two minor peaks in July and September, respectively, similar to the pattern of the seasonal change of the total phytoplankton cell abundance (Figure 2). The spatial change of both DiPPR and phytoplankton 18S rDNA gene abundances showed a weak decreasing pattern from the western Sound to the central and eastern Sound, with large variation within each station in each month (Figure 3C and Figure S2B,C). This pattern was similar to that of phytoplankton cell abundance (Figure 2) in LIS. However, DiPPR gene abundance showed no significant correlations with temperature, salinity, nutrient concentration, and chlorophyll *a* (Figure 4, Figure 5 and Figure 6).

When DiPPR gene abundance was normalized to the monthly average dinoflagellate cell abundance, peaks appeared at stations E1 and J2-K2 (4.00 × 10^3^, 3.46 × 10^3^, 6.82 × 10^3^ copies/cell, respectively; Figure 3D). The highest levels of DiPPR and phytoplankton 18S rDNA gene abundance were both at station A4 (2.17 ± 3.18 × 10^5^ copies·mL^−1^ and 10.07 ± 9.55 × 10^6^ copies·mL^−1^, respectively), with the lowest at station H4 (0.30 ± 0.36 × 10^5^ copies·mL^−1^ and 3.72 ± 4.35 × 10^6^ copies/mL^−1^, respectively; Figure 3C and Figure S2B). Annual average gene abundances were 4.2–21.7 × 10^4^ copies·mL^−1^ and 4.03–10.07 × 10^6^ copies·mL^−1^ in western Sound stations (A4 to F2), 3.0–4.9 × 10^4^ copies·mL^−1^ and 3.72–5.03 × 10^6^ copies·mL^−1^ in the central Sound (H4, I2), and 4.1–6.4 × 10^4^ copies·mL^−1^ and 2.80–4.36 × 10^6^ copies·mL^−1^ in the eastern Sound (J2, K2), respectively, for DiPPR and phytoplankton 18S rDNA (Figure 3).

### 3.3. Correlation between DiPPR/18S rDNA Gene Abundance and Environmental Factors

The relationship of DiPPR and 18S rRNA gene abundances with environmental factors is shown in Figure 4, Figure 5 and Figure 6. DiPPR gene abundance was not clearly correlated with the concentration of ambient nitrogen nutrients (ammonia, nitrate + nitrite, and total dissolved nitrogen), dissolved phosphorus, chlorophyll *a*, or ambient water temperature. However, DiPPR copy number showed a weak negative linear correlation with orthophosphate concentration (two-way ANOVA df = 1, F = 5.896; R^2^ = 0.060, *p* < 0.05) and salinity (df = 1, F = 15.769; R^2^ = 0.145, *p* < 0.01). On the other hand, phytoplankton18S rDNA gene abundance was not clearly correlated with ammonia concentration, salinity, or ambient water temperature; however, a strong linear positive relationship with chlorophyll *a* concentration (df = 1, F = 164.187; R^2^ = 0.638, *p* < 0.01), and a weak negative correlation with the concentration of nitrate + nitrite (df = 1, F = 10.135; R^2^ = 0.098, *p* < 0.01), total dissolved nitrogen (df = 1, F = 6.354; R^2^ = 0.0640, *p* < 0.05), orthophosphate (df = 1, F = 11.994; R^2^ = 0.114, *p* < 0.01), and dissolved phosphorus (df = 1, F = 6.595; R^2^ = 0.0662, *p* < 0.05) was demonstrated (Figure 4, Figure 5 and Figure 6).

### 3.4. Correlation between DiPPR/18S rDNA Gene Abundance and Total Phytoplankton Cell Abundance

The correlation of DiPPR/18S rRNA gene abundances with total community phytoplankton cell abundance is shown in Figure 6(C1). DiPPR gene abundance did not show a clear correlation with community cell abundance; however, phytoplankton18S rDNA copy number showed a positive correlation with total community phytoplankton cell abundance (df = 1, F = 39.039; R^2^ = 0.295, *p* < 0.05).

### 3.5. DiPPR Gene Diversity in LIS

**Figure 7:**
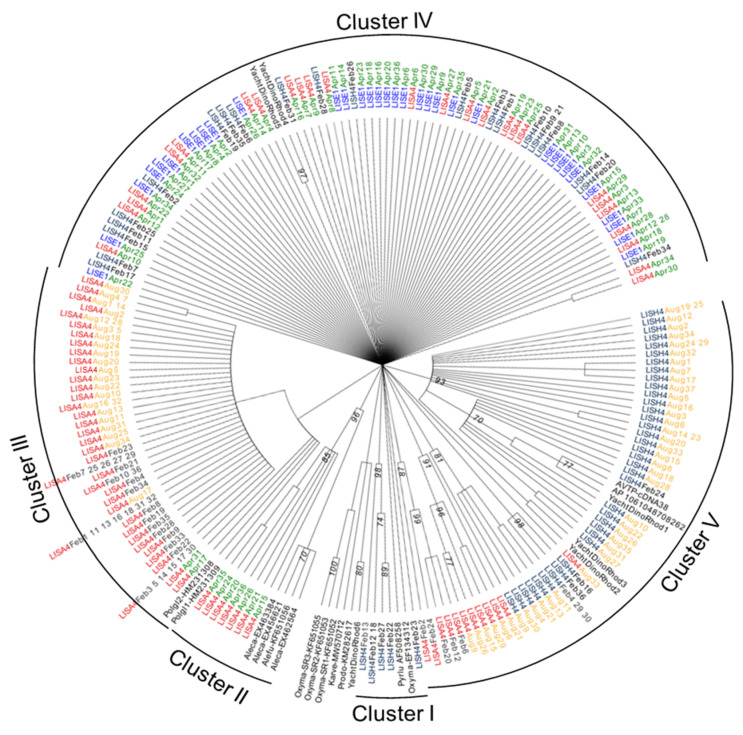
Maximum Likelihood tree of dinoflagellate rhodopsin genes obtained in this study. The representatives of the dinoflagellate rhodopsin genes reported in GenBank are included.

Two hundred and nine sequences were obtained from the genomic DNA isolated from six water samples, i.e., A4 in February, April, and August, E1 in April, and H4 in February and August (Table S2). These sequences were used in the diversity analysis along with the representatives of the reported dinoflagellate rhodopsin sequences as well as the six full-length rhodopsin sequences obtained in this study (YachtDinoRhod1, 2, 3, 4, 5, and 6; Figure 7). Most of the sequences were 306 bp long, excluding the primers; however, in most A4 February clones (19 out of 24), there was a 2-nt insertion in the sequence, resulting in frameshift; therefore, these sequences are considered rhodopsin pseudogenes.

Eighty-five (41%) of the sequences had unique putative amino acid sequences, indicating that the DiPPR gene is diverse in LIS. The most diverse samples were from A4 and E1 in April (74% and 76% of the amino acid sequences were unique, respectively; Figure 7). The least diverse sequences were from stations A4 and H4 in February (25% and 36% unique, respectively).

Phylogenetic analyses revealed that the sequences could be grouped into five clusters (Figure 7). In Cluster I, there were six sequences (two identical), all of which were from the Station H4 February water sample. These sequences were closest to *O. marina* PPR.

Cluster II had eight sequences solely from water samples at Station A4 in April and were tightly grouped with *Alexandrium* PPR (Figure 7).

Cluster III was composed of sequences solely from the Station A4 water samples in February (36 sequences), April (1) and August (26). These sequences were closely related to *Polarella glacialis* PPRs.

Cluster IV contained sequences from all three tested stations in February and April, including A4 in April (26), E1 in April (34), and H4 in February (21). These sequences are grouped with the other two full-length dinoflagellate proton-pump rhodopsins obtained in this study. In this cluster, sequences from different stations in the same month or the same station in different months could be clustered together, suggesting that some similar DiPPRs, and likely from similar dinoflagellate taxa, exist throughout the Sound all year round.

Cluster V consisted of sequences mainly from the Station H4 August water sample (37), but some from H4 February (6) and A4 August water samples (7). These sequences were clustered together with the PPRs from unknown dinoflagellates we obtained in the previous study and three full-length PPRs we isolated from the water samples near our laboratory (Figure 7; see next section for details).

### 3.6. Full-Length cDNAs of DiPPRs from the Field Water Sample and K. veneficum

Six full-length DiPPR cDNAs from the water sample collected at our campus (YachtDinoRhod1 to 6) and two full-length rhodopsin cDNAs from *K. veneficum* were deposited to GenBank with Accession # MW570706-MW570713. YachtDinoRhod1 shared high nt and aa similarities with the two sequences we obtained previously from the water sample at a similar location (AVTP-cDNA38 GU553957 and AP_1061048708262 GU554267; Figure 7; [8]). YachtDinoRhod2 and 3 are clustered with the sequences obtained from Station H4 in August. YachtDinoRhod4 and 5 are grouped with many sequences from water samples collected in all three stations (Figure 7). YachtDinoRhod6, however, had a distinct sequence from the other five sequences, sharing some similarity with *P. minimum* rhodopsin yet with low bootstrap support.

## 4. Discussion

Rhodopsin-based phototrophy is now recognized as an important mechanism of solar energy capture in the marine ecosystem [3,4,6,9,10]. By measuring all-*trans* retinal, chlorophyll *a*, and bacteriochlorophyll *a* abundance in the Mediterranean Sea and the Atlantic Ocean, Gómez-Consarnau and colleagues discovered that proton-pump proteorhodopsin-based phototrophy might contribute the same amount of light energy fixation as chlorophyll *a*-based phototrophy does, producing energy sufficient to support basal metabolism of bacteria in the surface ocean [7]. However, this method cannot distinguish whether the proton-pump proteorhodopsin-based phototrophy is from bacteria, picodinoflagellates, or other picoeukaryotes. Using metatranscriptomics coupled with the 18S rDNA tag sequencing method, Vader et al. [5] examined the expression of microbial proton-pumping rhodopsins and other genes in the mid-summer for function and composition of marine protists (size 0.45–10 μm) in the high-Arctic Billefjorden. While we were preparing this study for submission, a recent publication based on a whole-assemblage metatranscriptomics study using the Illumina high-throughput method investigated the diversity and expression dynamics of PPR in microbial eukaryotes and prokaryotes at a continental shelf and a slope site in the northern South China Sea [36]. No study, however, has been dedicated to understanding the seasonal variation of DiPPR gene abundance in the field. The present study provides the first documentation of the seasonal distribution pattern of the DiPPR gene in the LIS estuary off the Atlantic Ocean.

### 4.1. Dinoflagellate Specific PPR Primers

We designed primers at the conserved regions of known typical DiPPR (Table 2). Most of the reported DiPPRs, including those from *P. lunula* [18], *O. marina* [8,10,19,20], *P. glacialis* [8], and *A. catenella* [10], belong to proton-pump rhodopsin from *r*-proteobacteria, and share > 70% nt and > 80% aa similarities. The more recently reported rhodopsin sequences from *Prorocentrum donghaiense* [21] and *Karlodinium veneficum* ([22] and this study), however, were not included in the alignment for primer designing. These sequences are very distinct, sharing < 45% of aa similarity with typical DiPPR (Figure 7). Likely, the DiPPR primers we designed did not amplify rhodopsin genes for *Prorocentrum* and *Karlodinium* species in the field, even though both species occur in LIS [37,38]. When testing the DiPPR primers for both the cDNA and gDNA of the phytoplankton species listed in Table 1, we discovered that for some tested dinoflagellates, i.e., *Alexandrium pacificum*, *Pyrocystis lunula*, *P. noctiluca*, and *Symbiodinium microadriaticum*, only cDNA gave positive amplification. This might be due to the existence of intron(s) in the targeted PCR region, resulting in unsuccessful amplification of DiPPR from gDNA. No amplification appeared for any of the non-dinoflagellate algae tested, indicating these primers are dinoflagellate specific or that those other algae do not possess PPR. Also, these primer sets did not amplify any sensory-type rhodopsins when cloned cDNA of *O. marina* sensory-type rhodopsins [10] were used as templates. As such, the present study provides a typical DiPPR-specific primer set for future use in investigating the diversity and abundance of dinoflagellate PPR in natural environments.

### 4.2. Spatial and Seasonal Changes of Dinoflagellate PPR Gene Abundance in Long Island Sound

With the DNA extracted from a year’s worth of samples (95 in total), 18S rDNA qPCR successfully amplified 18S rDNA from each sample, and its copy numbers exhibited a similar dynamic pattern to the total phytoplankton cell abundance from microscopic counts (Figure 3, Figure 4 and Figure 6(C2) and Figure S2(A2,C2)), and corresponded well with chlorophyll *a* concentration in each sample (Figure 5(C2)), with an exception in the September samples where there was a diatom peak yet only minor increase of 18S rDNA copy number. This inconsistency could be due to the bloom diatom species harboring a low amount of 18S rDNA copies in the genomes [39]. This verifies that the DNA quality and PCR conditions in this study were reliable. From these DNA samples, qPCR results showed that the monthly gene abundance of DiPPR varied markedly among stations (Figure 2 and Figure 3).

The temporal change of DiPPR abundance in LIS showed two major PPR abundance peaks, one in April (also high in March) and the other in July (Figure 3A). The July peak could correspond to the high average dinoflagellate cell abundance in this month. However, in April, total dinoflagellate cell abundance was rather low at most of the stations, yet DiPPR gene abundance was very high at stations A4, B3, and E1 (Figure S2). As a result, when DiPPR gene abundance was normalized to the monthly average dinoflagellate cell number, only the April peak remained (Figure 3B). When taking a closer look at the dinoflagellate species in March and April, we found that the dinoflagellate *Peridinium quinquecorne* Abe was abundant at stations E1, F2, H4, I2, J2, and K2 in March and A4, B3, C1, D3, E1, and F2 in April. We are not certain whether this dinoflagellate was the major contributor to the high DiPPR gene abundance during these months because we do not have this species in culture to confirm its gene sequence. On the other hand, the high dinoflagellate cell abundance in July was largely contributed by *Prorocentrum triestinum* Schiller, which most likely has similar rhodopsin with *P. shikokuense* and would not be detected with our DiPPR qPCR primers. This may explain why DiPPR abundance per dinoflagellate cell was low in July.

Spatially, DiPPR gene abundance showed a weak trend of being greater in western Sound than in central and eastern Sound (Figure 3C). However, when normalized to the monthly average dinoflagellate cell abundance, DiPPR gene abundance showed peaks at stations E1, J2, and K2 (Figure 3D and Figure S2). It is important to note that inclement weather prevented sampling at stations J2 and K2 for several months (4 months and 5 months, respectively) in 2010 (Table S1, Figure S2); therefore, abundance data could be biased for these stations. In addition, the peak at station E1 was primarily caused by the high DiPPR copy number in some species in April, possibly contributed by *P. quinquecorne* and a generally low abundance of dinoflagellate cell numbers in this station.

We did not observe a clear correlation between DiPPR gene abundance and any particular environmental factor other than weak negative correlations with orthophosphate concentration and salinity (Figure 4, Figure 5 and Figure 6). In April, when the DiPPR gene copy number was the highest, the values of all the measured environmental factors (e.g., salinity, water temperature, chlorophyll *a*, the concentration of ammonia, nitrate + nitrite, orthophosphate, total dissolved nitrogen, and total dissolved phosphorus) were low in general. Similarly, in July, when the DiPPR gene copy number was also high, all of the environmental factors except water temperature were low. This result suggests that DiPPR gene abundance in LIS is not influenced by water temperature or nitrogen nutrient concentration. The observed negative correlation of DiPPR gene abundance with salinity is interesting, as it suggests that the DiPPR-harboring dinoflagellates might be slightly favored by lower salinities. In addition, the negative correlation of DiPPR abundance with phosphate is reminiscent of recent findings that PPR gene expression was upregulated under phosphorus-stressed conditions in *P. donghaiense*, in which DiPPR is postulated to facilitate the dinoflagellate to endure or thrive under phosphate deficiency [21,23]. The functional association of DiPPR with phosphorus nutrition warrants further investigation in the future.

The seasonal variation in 18S rDNA gene abundances was similar to that of phytoplankton chlorophyll *a* concentrations and cell abundances (Figure 5(C2)) The strong positive relationship between phytoplankton 18S rDNA gene abundance and chlorophyll *a* concentration (R^2^ = 0.638, *p*-value < 0.01; Figure 5) indicates that our quantitative Real-Time PCR system worked properly, and the 18S rDNA abundance data are reliable proxies of phytoplankton cell abundance in a community. The weak negative correlations of 18S rDNA copy number with orthophosphate and nitrogen (other than ammonia) concentrations might be due to some of the nitrogen and phosphorus nutrients being consumed by phytoplankton in their growth.

### 4.3. DiPPR Gene Diversity in LIS

To explore the diversity of dinoflagellate PPR genes in LIS, we chose six DNA samples for cloning and sequencing of the dinoflagellate PPR genes, including three samples from A4 in February, April, and August, one sample from station E1 in April, and two samples from station H4 in February and August. These samples were chosen to obtain representatives to investigate the diversity of dinoflagellate rhodopsin by location in LIS (western vs. central) and by season (winter, spring, and summer). In addition, there was a peak of DiPPR abundance in April, and we intended to explore what kind of dinoflagellates they were.

The 209 sequences obtained could be grouped into five clusters, but none of them were identical to the few reported DiPPR genes (Figure 7); therefore, we cannot attribute them to specific dinoflagellate species. Cluster I contained six sequences solely from the Station H4 February water sample that was somewhat close to *O. marina* PPR, Cluster II were sequences from the water sample at Station A4 in April that were similar to *Alexandrium* PPR, while Cluster III composed of sequences from Station A4 water samples in all the investigated months that were grouped with *P. glacialis* PPR. Cluster IV contained sequences from all three tested stations in February and April but not in August, while Cluster V had sequences mostly from Station H4 in August and some from H4 in February and A4 in August. These results indicate that some dinoflagellates exist only at a certain time of year at a specific location in the Sound, while others can be found at stations all year round. The functions and ecological implications of the DiPPR diversity pattern remain to be uncovered.

## 5. Conclusions

In conclusion, we isolated six novel full-length dinoflagellate proton-pump rhodopsin cDNAs, augmenting the DiPPR database that is crucial to PPR research. We also developed primers with verified efficiency and specificity, which will be useful for future studies on DiPPR in natural protist assemblages. This study represents the first reported effort, to the best of our knowledge, of using quantitative PCR to analyze the spatial and temporal variations of the abundance and diversity of DiPPR genes over the course of one year in a natural marine ecosystem. In addition, the results provide clues for future research to understand the ecological role of DiPPR with respect to phosphorus nutrition and population growth of dinoflagellates.

## Figures and Tables

**Table 1:** Stations and months of water samples collected in this study.

Month	Station	Sample #	Month	Station	Sample #	Month	Station	Sample #
January	A4	1		C1	33		F2	65
	B3	2		D3	34		H4	66
	C1	3		E1	35		I2	67
	D3	4		F2	36	October	A4	68
	E1	5		H4	37		B3	69
	F2	6		I2	38		C1	70
	H4	7		K2	39		D3	71
	I2	8	May	A4	40		E1	72
	J2	9		B3	41		F2	73
	K2	10		C1	42		H4	74
February	A4	11		D3	43		I2	75
	B3	12		E1	44		J2	76
	C1	13		F2	45		K2	77
	D3	14		H4	46	November	A4	78
	E1	15		I2	47		B3	79
	F2	16		J2	48		C1	80
	H4	17		K2	49		D3	81
	I2	18	July	A4	50		E1	82
	J2	19		B3	51		F2	83
	K2	20		D3	52		H4	84
March	A4	21		E1	53		I2	85
	B3	22		F2	54		J2	86
	C1	23		H4	55	December	A4	87
	D3	24	August	A4	56		B3	88
	E1	25		B3	57		C1	89
	F2	25		C1	58		D3	90
	H4	27		D3	59		E1	91
	I2	28		F2	60		F2	92
	J2	29		H4	61		H4	93
	K2	30	September	B3	62		I2	94
April	A4	31		D3	63		J2	95
	B3	32		E1	64			

**Table 2:** Primers used in the present study.

Primer Name	Sequence Information	Reference
DinoRhodF1N	TACAATGCRCTSTCSTTCGGNATHGC	This study
DinoRhodF2N	CTCTCGTTCGGCATHGCNGCNATGGG	This study
DinoRhodF3N	ATGGGYTCYGCAACCRTYTTCTTYTGG	This study
SymkaLHC3R1	TCTCTCGAATTCCGTGTGCTTGTGAAACTTTTATC	This study
DinoRhodR3N	CTCACCNGGRTASCCCARNGCNACCAT	This study
DinoRhodR2N	CAGAGATCAAGACATGCTTCAGGAC	This study
DinoRhodR1N	ATGTACACYAANGGGTAAGTGMRCCA	This study
2-22RhodF1	GACCTTCATCGCAGCATACC	This study
2-22RhodF2	ATGGATTGGTTGCTGACAGTG	This study
YachtRhod1+2F	GCCATGGCCCCCCTTGC	This study
YachtRhod3F	GCCATGGCCCCACTTTCCG	This study
YachtRhod4+5F	GCCATGGCGCCTCTCCCTAC	This study
YachtRhod1Ra	TCAGCGGAGCAAACGGCCATC	This study
YachtRhod1Rb	CCAGCGGAGCAAACGGCCATC	This study
YachtRhod2Ra	TCACTGCAGGGAGCAGCCGC	This study
YachtRhod2Rb	CCACTGCAGGGAGCAGCCGC	This study
YachtRhod3Ra	TCAGCGCAACAAGCGGCC	This study
YachtRhod3Rb	CCAGCGCAACAAGCGGCC	This study
18SCOMF	TGCATGGCCGTTCTTAGTTGGTGG	[26]
18SCOMR	CACCTACGGAAACCTTGTTACGAC	[26]
DinoSL	DCCGTAGCCATTTTGGCTCAAG	[19]
DinoSL	DCCGTAGCCATTTTGGCTCAAG	[19]
Racer3	TGTCAACGATACGCTACGTAACG	Invitrogen
DinoSL	DCCGTAGCCATTTTGGCTCAAG	[19]
Racer3	TGTCAACGATACGCTACGTAACG	Invitrogen
KarveRhodF1	CGATTTCTTTCTGGATAATCTCCATT	This study
KarveRhodF2	GTTCACTACTTCTACATGCGTGAG	This study
KarveRhodF3	TACATGCGTGAGTTCTGGGT	This study
KarveRhodR1	TCACCCATGAAGATCTCAAACAAGAT	This study
KarveRhodR2	CAATGGGTAAATGCACCAACCAA	This study
KarveRhodR3	TCTGCCAAGTCATACGCCAAGTT	This study

**Table 3:** Phytoplankton species tested for dinoflagellate rhodopsin primers.

Phytoplankton Species	Strain	cDNA *	gDNA *
**Dinoflagellates**			
*Alexandrium catenella* (Whedon and Kofoid) Balech (formally *A. fundyence* Balech)	CCMP1719	+	+
*Alexandrium pacificum* Litaker	ACHK	+	−
*Amphidinium carterae* Hulburth	AC	+	+
*Fugacium kawagutii (formerly Symbiodinium kawagutii)*	CCMP2468	−	−
*Heterocapsa triquetra* (Ehrenberg) Stein	CCMP 448	−	−
*Karenia brevis* (Davis) Hansen and Moestrup	Wilson	−	−
*Karlodinium veneficum* Larsen	CCMP1975	−	−
*Oxyrrhis marina* Dujardin	CCMP1795	+	+
*Pfiesteria piscicida* Steidinger and Burkholder, 1996	CCMP1831	−	−
*Pfiesteria shumwayae* Glasgow and Burkholder	CCMP2359	−	−
*Polarella glacialis* Montresor, Procaccini, and Stoecker	CCMP2088	+	+
*Prorocentrum shikokuense* Hada (=*P. donghaiense* Lu*)*		−	−
*Prorocentrum minimum* (Pavillard) Schiller	CCMP696	−	−
*Pyrocystis lunula* Schütt		+	−
*Pyrocystis noctiluca* Murray ex Haeckel	CCMP732	+	−
*Symbiodinium microadriaticum* Freudenthal	CCMP 2458	+	−
*Symbiodinium* sp.	SSB01	−	−
**Non-dinoflagellates**			
*Ditylum brightwellii* (T. West) Grunow	CCMP 2227	−	−
*Dunaliella tertiolecta* Butcher	CCMP1320	−	−
*Emiliania huxleyi* (Lohmann) Hay and Mohler		−	−
*Heterosigma akashiwo* Hada ex Hara and Chihara	CCMP 452	−	−
*Odontella sinensis* (Greville) Grunow	CCMP 1815	−	−
*Rhodomonas* sp.	CCMP768	−	−
*Skeletonema costatum* (Greville) Cleve	China30	−	−
*Thalassiosira pseudonana* Hasle and Heimdal	CCMP1335	−	−

* Note: Three sets of primers (DinoRhodF1N- DinoRhodR1N, DinoRhodF2N-DinoRhodR2N, and DinoRhodF3N-DinoRhodR3N) were used in the test; “+” means DiPPR was detected by at least one set of the primers and the PCR products were proven to be DiPPR by direct sequencing, “−” means none of the primer sets gave positive amplification of DiPPR.

## Data Availability

The nucleotide sequences of the field dinoflagellate proton-pump rhodopsin obtained in this study are available in Supplementary Table S2.

## Supplementary Materials

The following supporting information can be downloaded at: https://www.mdpi.com/article/10.3390/microorganisms12030628/s1, Figure S1: Monthly measurement of environmental factors in 2010, Long Island Sound. (A) Monthly changes of Chlorophyll *a*, temperature, and salinity. (B) Monthly changes of concentration of ammonium, nitrate + nitrite, orthophosphate, total dissolved nitrogen, and total dissolved phosphorus in 2010 Long Island Sound. (C) Monthly changes of biogenic silica and dissolved silica concentration in 2010, Long Island Sound; Figure S2: Seasonal changes of phytoplankton cell abundance (A), dinoflagellate rhodopsin (B), phytoplankton 18S rDNA abundance (C), and the relative ratio of gene abundance of dinoflagellate rhodopsin vs. phytoplankton 18S rDNA (D) in 2010, Long Island Sound; Table S1: 2010 Long Island Sound phytoplankton cell count for 10 stations; Table S2: 209 dinoflagellate proton-pump rhodopsin sequences obtained from the six water samples including A4 in February, April, and August, E1 in April, and H4 in February and August.
